# CerealsDB 3.0: expansion of resources and data integration

**DOI:** 10.1186/s12859-016-1139-x

**Published:** 2016-06-24

**Authors:** Paul A. Wilkinson, Mark O. Winfield, Gary L. A. Barker, Simon Tyrrell, Xingdong Bian, Alexandra M. Allen, Amanda Burridge, Jane A. Coghill, Christy Waterfall, Mario Caccamo, Robert P. Davey, Keith J. Edwards

**Affiliations:** School of Biological Sciences, University of Bristol, Bristol, BS8 1UG UK; The Genome Analysis Centre (TGAC), Norwich Research Park, Norwich, UK; National Institute of Agricultural Botany (NIAB), Huntingdon Road, Cambridge, UK

**Keywords:** Wheat, Single nucleotide polymorphisms, SNPs, Database, CerealsDB, Genomic selection

## Abstract

**Background:**

The increase in human populations around the world has put pressure on resources, and as a consequence food security has become an important challenge for the 21st century. Wheat (*Triticum aestivum)* is one of the most important crops in human and livestock diets, and the development of wheat varieties that produce higher yields, combined with increased resistance to pests and resilience to changes in climate, has meant that wheat breeding has become an important focus of scientific research. In an attempt to facilitate these improvements in wheat, plant breeders have employed molecular tools to help them identify genes for important agronomic traits that can be bred into new varieties. Modern molecular techniques have ensured that the rapid and inexpensive characterisation of SNP markers and their validation with modern genotyping methods has produced a valuable resource that can be used in marker assisted selection. CerealsDB was created as a means of quickly disseminating this information to breeders and researchers around the globe.

**Description:**

CerealsDB version 3.0 is an online resource that contains a wide range of genomic datasets for wheat that will assist plant breeders and scientists to select the most appropriate markers for use in marker assisted selection. CerealsDB includes a database which currently contains in excess of a million putative varietal SNPs, of which several hundreds of thousands have been experimentally validated. In addition, CerealsDB also contains new data on functional SNPs predicted to have a major effect on protein function and we have constructed a web service to encourage data integration and high-throughput programmatic access.

**Conclusion:**

CerealsDB is an open access website that hosts information on SNPs that are considered useful for both plant breeders and research scientists. The recent inclusion of web services designed to federate genomic data resources allows the information on CerealsDB to be more fully integrated with the WheatIS network and other biological databases.

**Electronic supplementary material:**

The online version of this article (doi:10.1186/s12859-016-1139-x) contains supplementary material, which is available to authorized users.

## Background

A rapidly expanding human population has meant that food security has become one of the primary global concerns that scientists and governments are seeking to address. Cereal crops are a major source of nutrition for both humans and domesticated animals. The development of novel varieties that result in an increase in yield, and have other beneficial characteristics such as disease and drought resistance, should help to safeguard the future supply of these economically important crops.

Single nucleotide polymorphisms (SNPs) have become the marker of choice used by plant breeders in marker assisted selection (MAS). The CerealsDB website was developed in response to a need to collate and present validated SNP data for wheat (*Triticum aestivum*) that is easily accessible to wheat breeders and researchers around the world.

CerealsDB was originally created in 2003 to host a dataset of 26,382 EST sequences [1]. In 2010 it was expanded to include a database of DArT markers, SNPs validated using the KASP genotyping platform and pages that allowed users to download the draft genome of the wheat variety ‘Chinese Spring’ [2]. This version 2.0 of the CerealsDB website was also extensively redesigned using cascading style-sheets (CSS) to standardise the format of the site, and improve its presentation, to provide a more intuitive browsing experience. The enduring ethos of CerealsDB has been to implement a website that is simple to use, which displays the data in a format that is easy to understand and to release the data into the public domain. CerealsDB has maintained a policy of making its data freely available to the public without restrictions or intellectual property rights. The site does not require registration, access is not password protected and data is provided to the wheat breeding and research communities as quickly as possible to maximise the potential utility of the data.

Since the release of CerealsDB version 2.0 there has been another extensive redesign of the site to accommodate the addition of new SNP datasets that were validated on various platforms: the Illumina iSelect platform; the 820 K Axiom HD Wheat Genotyping Array and 35 K Axiom Wheat Breeders Array, that were developed in collaboration with Affymetrix [3]; and Taqman. The website and database was also migrated to a new server in response to the increase in the number of users and volume of data hosted by the website.

The latest incarnation of CerealsDB (version 3.0) contains links to functional SNPs which are defined as those SNPs with a high probability of affecting phenotype *i.e.* those non-synonymous variants that lie within protein-coding regions and have a significant effect on the function of the protein. These SNPs are of interest to breeders as perfect markers for traits and to academics as candidate genes.

In addition, version 3.0 has employed the use of web services to expose the underlying data in the CerealsDB database in formats that enable integration of its data with other online genomic data resources. The CerealsDB web services have been developed in collaboration with partners from the Wheat Initiative [4] Wheat Information System [5] (WheatIS) expert working group to produce an application programming interface (API) that is interoperable and conforms to a set of common data standards.

## Construction and content

### Implementation

CerealsDB version 3.0 uses a MySQL relational database management system (RDMS) database (version 14.14) running on a Linux server (Ubuntu OS version 12.04) and hosted using the Apache2 web server (version 2.4.7) with Perl and PHP scripts used for all data retrieval and output. The site was migrated to a new server that has significantly more resources than the previous web server (CerealsDB version 2.0), with 256 GB RAM, 32 CPU’s and 2.6 TB of disk space. The migration of the site to a new server was considered essential as the amount of data hosted and the number of users accessing it (average of 13,300 per month—see Additional file 1: Figure S1.) meant that the original web server was no longer fit for purpose.

The CerealsDB database has experienced a rapid increase in size from 11 tables in version 2.0, to 35 tables in version 3.0 (see Additional file 1: Figure S2.), with more than a ten-fold increase in the amount of data stored in the database (CerealsDB contained 151 Mb of data, which has increased to 1.7 Gb in the latest version). When the tables were constructed, data types were selected that would result in a more efficient indexing to ensure a rapid response to queries. Fourteen of the tables share an identifying relationship with the ‘Contig’ table through their common primary key, ‘SNP_id’ (displayed in Additional file 1: Figure S2).

In addition to the ‘Contig’ table described previously [2], which holds 111,442 SNPs validated on the KASPAR genotyping platform [6], there are now SNPs that have been analysed using the iSelect, Axiom and Taqman genotyping platforms. Of the 81,587 SNPs that were validated using the wheat 90 K iSelect assay [7] there were 43,999 that have been mapped and 41,704 mapped unambiguously. The iSelect SNPs are located in the table called ‘iselect_all’ which also contains genotyping information on 252 wheat varieties, close relatives and progenitor species.

Two tables in the database relate to the 35 K wheat breeders array ‘axiom35’ and the 820 K array ‘axiom820’ described in Winfield et al*.* 2015. The ‘axiom35’ table contains 35,143 markers (of which 35,042 have been mapped). The ‘axiom820’ contains 819,571 SNPs, and 164,250 of these have been mapped (120,730 mapped unambiguously).

Taqman SNPs are stored in a table called ‘taqman_SNPs_mapped’ which contains 4800 entries, of which 4735 have been mapped. TaqMan Assays are a qPCR genotyping application developed by Thermo Fisher and based on 5’ nuclease chemistry that uses specific fluorescent probes to discriminate SNP variants. A separate table ‘taqman_core_set’ contains 935 SNPs that are known to be evenly distributed across the wheat genome.

The table called ‘funcSNPs’ contains 19,641 non-synonymous SNPs of which 2738 have been characterised as having a major effect on the protein function. Functional SNPs are defined as those with a FATHMM score less than -3.0 or greater than 3.0.

The largest table in the database is called ‘axiom820dataab’ consisting of 476 rows linking the axiom 820 K SNP codes to the genotyping data for 475 varieties of wheat and closely related species such as the A,B and D genome progenitors (Triticae and *Aegilops)*, and more distantly related species such as *Secale* (Rye) and *Thinopyrum*.

Alignment information is contained in three tables called ‘snps_vs_brachy’ (which are segregated by the BLAST alignment evalue) and the ‘burdock’ table. These tables map SNPs to the fully contiguous *Brachypodium distachyon* genome and also contain physical map locations for three mapping populations (Avalon × Cadenza, Savannah × Rialto, Synthetic × Opata).

### Data sources

SNP data were included from previous studies [1, 8, 9] as well as from ongoing experiments. SNPs were validated using the LGC Kompetitive Allele‐Specific PCR (KASPar), Illumina iSelect, Affymetrix axiom and Thermofisher Taqman genotyping platforms. All SNP data were curated to remove redundancy and duplicate values. The contigs used in the database were generated from an assembly of wheat (5x Chinese Spring var.) cDNA sequences and also obtained from the International Wheat Genome Sequencing Consortium (IWGSC) which used flow sorted chromosomes to generate a high quality reference genome.

The CerealsDB database currently consists of data on 1,017,400 SNPs characterised on at least one of the four genotyping platforms, of which 86,514 (8.5 %) have been mapped to a genetic position. These SNPs are uniquely identified by a 10-character code, beginning with a Bristol SNP (BS) prefix, followed by 8 digits (*e.g*., BS00000001). For the axiom SNPs unique IDs were created for each axiom probe using an 11-character code, beginning with an ‘AX-‘ prefix followed by 8 digits (e.g., AX-94381124). Users can enter this code on the CerealsDB site to retrieve information about the SNP, such as its position within a contig, the contig sequence, map location and adjacent SNPs. Links are also provided to the Ensembl Plants database [10] if the SNP is found to be mapped to the current wheat genome reference.

## Utility and discussion

### Web interface and user querying capabilities

**Fig. 1:**
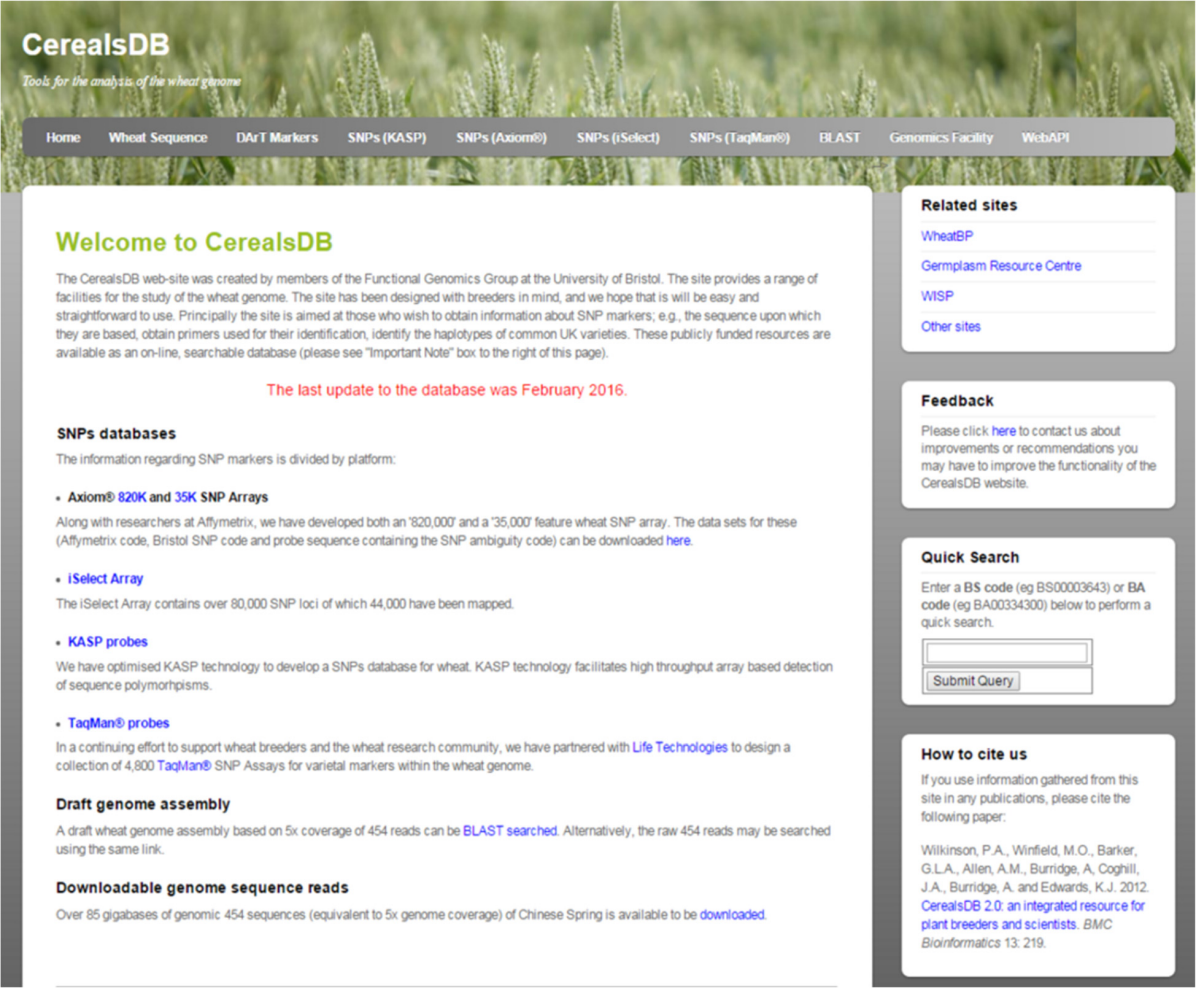
The latest home page for CerealsDB showing a completely redesigned website, with a horizontal navigation bar at the top that links to separate areas of the site for Wheat sequences, Dart markers, KASP SNPs, axiom SNPs, iSelect SNPs and Taqman SNPs. There is also a link to the BLAST databases and pages describing the web services available on CerealsDB. Additional navigation menus are available on the right hand side of the website including a quick search facility for SNPs with an axiom or BS code

The CerealsDB website contains a range of datasets that are accessible via the horizontal menu bar at the top of each page which links to SNPs validated on one of four genotyping platforms (KASP, Axiom, iSelect and Taqman) see Fig. 1. A BLAST [11] search page is also available that allows users to query a range of databases that include the Brenchley [12] and Chapman [13] wheat genome assemblies, the Rye exome assembly and validated SNPs. A drop down field is available for setting the e-value cut-off.

There are also links to a download page for the draft wheat genome, a wheat EST database, a search page for wheat DArT data and a page describing the mapping of DArT markers using wheat deletion lines.

The SNP database allows SNP name and contig queries using a BS code or Axiom code. Some queries (*e.g.* SNP name and Axiom code) are exact. The initial page for each of genotyping platforms contains statistics on the SNPs for that particular platform. All SNPs listed in CerealsDB have been assigned a unique ID and the output page for a query gives information about the gene name and locus. As described previously [1], chromosome information can be accessed via a clickable image (ideogram).

Wheat ESTs can be searched by gene name, homology search (via BLAST) or westdb cloneID. The wheat DArT markers can be searched by marker name or wheat line and the facility is available to download the DArT datasets in Excel format. Mapped DArT markers can also be viewed on their respective chromosome by clicking the appropriate chromosome image of the ideogram.

**Fig. 2:**
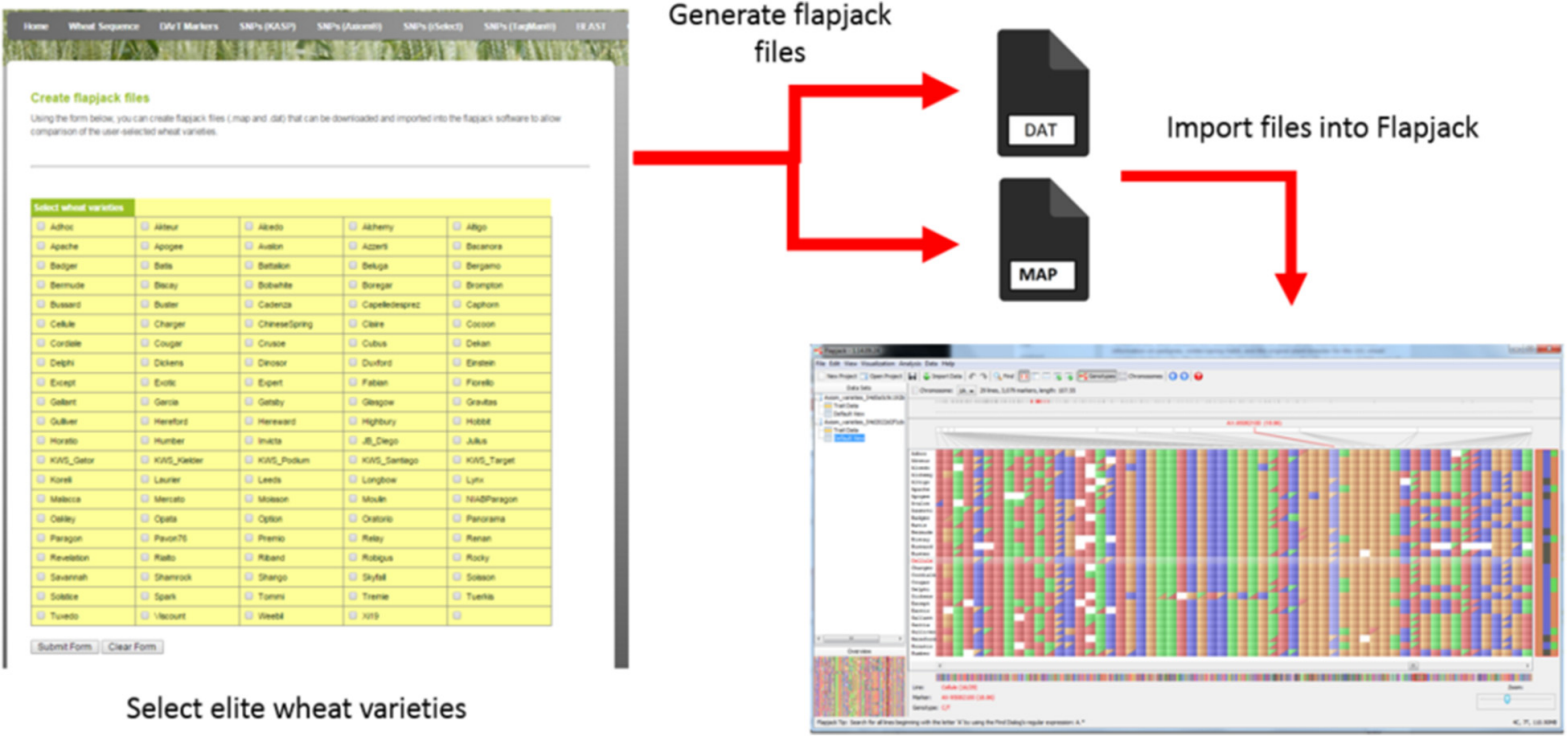
The diagram shows the generation of flapjack .map and .dat files from axiom genotyping data. The user selects one of three physical maps and then a selection of wheat varieties (105 available). The resulting flapjack formatted files can then be visualised with the Flapjack software

Users can create Flapjack files based on the axiom genotyping data (.map and .dat) that can be imported into the flapjack software to allow comparison of the user-selected wheat varieties (see Fig. 2). Flapjack is a multi-platform application providing interactive visualisations of high-throughput genotype data, allowing for rapid navigation and comparisons between lines, markers and chromosomes [14].

### Web services

A web service can be described as a collection of open protocols and data standards that allow data to be exchanged between software applications or computer systems. These applications can be implemented in a wide variety of programming languages and run on a diverse set of platforms. Web services exchange data over local or global computer networks and through the use of consistent data interchange formats such as JSON, can enable interoperability (*i.e.* data can be exchanged between different programming languages such as Perl and Python, or different software that may run on different operating systems such as Windows and Linux). JSON is a text-based open standard for data exchange that has been designed to be human-readable and easy for machines to parse and create. JSON uses a simple methodology to store information as key-value pairs, facilitating the structuring of data so that it can be consumed by other services. For the web services we adopted a JSON-based schema for messaging between the CerealsDB server and its clients.

The CerealsDB web services use HTTP methods to implement the concept of REST architecture. A RESTful web service will typically define a URI (Uniform Resource Identifier), and provides resource representation such as JSON or XML and a set of HTTP Methods. The web services that have been implemented on CerealsDB are based on the WheatIS Grassroots API to ensure that we share common nomenclature with the wider community. The Grassroots API is able to expose data and analytical services through a consistent descriptive specification and industry-standard software packages, so that when two or more data resources use the Grassroots API, they can easily be federated together and enable a single view over these multiple disparate resources.

**Fig. 3:**
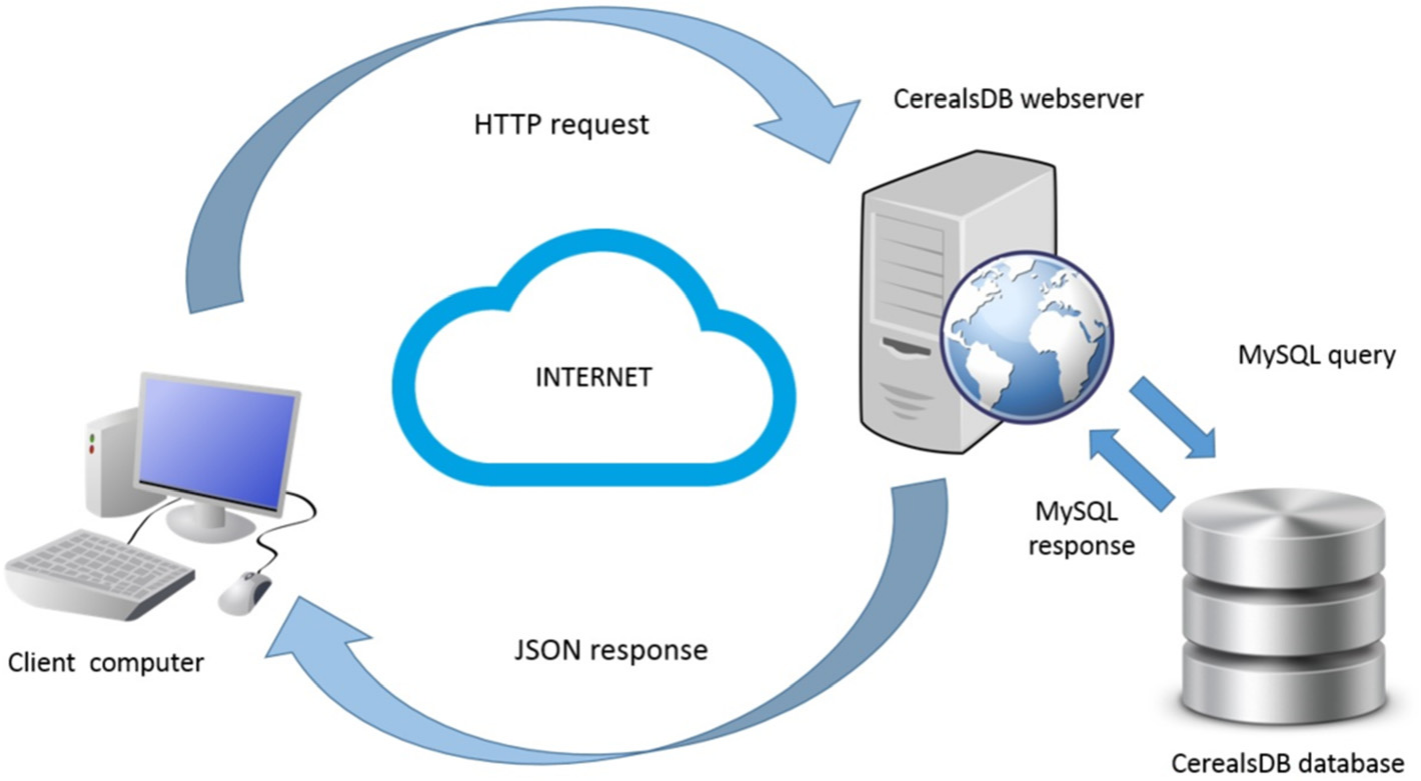
The diagram shows a simple request to CerealsDB made by a client computer across the web in the form of an HTTP request which contains information encoded in JSON. The CerealsDB webserver then interprets this request and queries the CerealsDB MySQL database. The response from the database is converted into JSON and returned to the client computer, where the information embedded in the JSON code can be parsed

Figure 3 shows a simple request made by a client computer across the web in the form of an HTTP request which contains information encoded in JSON. The CerealsDB webserver then interprets this request and queries CerealsDB. The response data is represented as a JSON fragment and returned to the client computer, where the information embedded in the JSON code can be parsed.

The available web services can be accessed by sending a JSON request to CerealsDB (via cURL or a perl script for example). The initial web services request to CerealsDB is described in the Additional file 1. This will return a JSON response containing a list of the services that the CerealsDB site provides (currently a Contig service and Search service). This response is also displayed in the Additional file 1, and users can parse this JSON code using JSON modules included with many popular programming languages, which contain JSON-specific decode/encode functions that can convert a JSON string into a data structure, and vice versa. Users can then construct a fresh JSON query to the web service to extract information on contig sequence that relates to a SNP, SNP position and other SNPs located on the same contig (an example of this is included in the Additional file 1).

The CerealsDB web services were written in Perl and example Perl and Python scripts are available to download from the website to help users construct queries. Additional examples of querying CerealsDB via the web services are provided on the site, describing the use of the cURL program on the command line and via a web browser’s address bar. These examples are available via the ‘WebAPI’ link in the horizontal menu bar.

### BLAST services

BLAST services on CerealsDB have been exposed to the WheatIS network using the Grassroots API [15] developed by The Genome Analysis Centre (TGAC). The Grassroots API is an architecture to allow both services and data to be shared between separate remote servers. A user connecting into a Grassroots server is able to access all of the services and data on any other connected Grassroots servers transparently without having to log into each server individually. All of the information is communicated using a standardised extensible schema. Connecting CerealsDB and the Grassroots servers based at TGAC means that, from the user’s perspective, they see a single Blast web service accessing many local databases.

### Functional SNPs

**Fig. 4:**
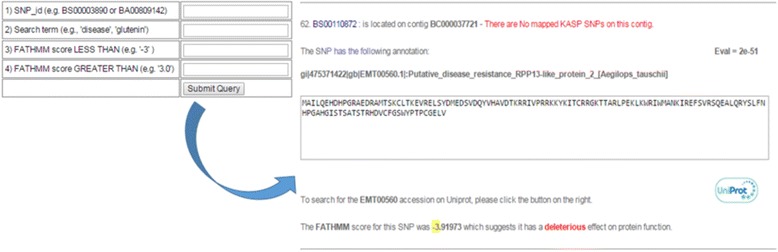
On the functional SNPs page it is possible to search for fSNPs and non-synonymous SNPs by name or a specific search term such as disease resistance. Functional SNPs can be selected by searching for SNPs with a FATHMM score less than -3.0 or greater than 3.0. Pressing the submit button generates a search and the resulting data is shown on the right of this diagram detailing the SNP amino acid sequence, BLAST annotations and FATHMM scores. If a SNP sequence has been annotated with a genbank accession number there will be an option to link out to the Uniprot website where additional information on gene ontologies, protein families and taxonomy can be found

A new search page that allows users to identify functional SNPs has been added to CerealsDB (see Fig. 4) and can be accessed from the following URL: http://www.cerealsdb.uk.net/cerealgenomics/CerealsDB/funcSNPs_select.php. Functional SNP prediction was performed using the functional analysis through hidden Markov models (FATHMM) technique described by Shihab et al*.* [16]. Users can search non-synonymous and functional SNPs by BS or axiom codes, search term (*e.g.* Disease resistance) or FATHMM score. The response from CerealsDB contains information on the Contig that contains the SNP, along with the translated amino acid sequence and the location of the amino acid change. If a BLAST annotation is available for this SNP it is displayed with the BLAST e-value, alongside the SNPs associated FATHMM score and an indication as to whether the SNP has an effect on the proteins function. If the amino acid sequence has been annotated with a genbank accession, a “Uniprot button” appears to the right of the screen, which links out to the Uniprot website [17] where users can find additional information on gene ontologies, protein families and taxonomy.

CerealsDB has evolved into a resource for researchers and plant breeders who are primarily interested in wheat SNPs. In addition to CerealsDB, there are a number of websites, such as Ensembl Plants [18], the URGI wheat portal website [19] and GrainGenes [20], that provide genomic information on wheat. Ensembl Plants hosts information on eleven plant species, including wheat, and incorporates the sequence data of Brenchley [12] with the International Wheat Genome Sequence Consortium’s Chromosome Survey Sequence [21], the Chapman genetic mapping data [13], as well as SNP markers that have been characterised by CerealsDB [2] and the Wheat HapMap project [22]. The site offers a comprehensive overview of wheat genomic resources with data being displayed in the Ensembl genome browser allowing users to search the genome sequence assembly, annotations of protein-coding regions and non-coding genes, and information on genetic variation and comparative genomics. Similarly, the URGI wheat portal maintains databases and tools that allow users to study genetic and genomic wheat data. The information hosted on the URGI site includes chromosome survey sequences, reference sequences, both physical and genetic maps, polymorphisms, phenotypic information and array data. The portal also provides links to international wheat projects and access to analysis tools such as annotation pipelines [19]. GrainGenes is another site focussed on a wide range of cereals such as wheat, barley, rye and oat genomes. It is a repository of information consisting of genetic maps, mapping probes and primers, genes, alleles and QTLs [20]. The site includes data on primer sequences, polymorphism descriptions, genotype and trait scoring data, experimental protocols, photographs of marker polymorphisms, disease symptoms and mutant phenotypes [20]. The three sites have been designed as ‘hubs’ that integrate genomic information from a wide range of sources and, as a result, it can be difficult for inexperienced users to navigate or access relevant information. CerealsDB differentiates itself from other sites by maintaining its primary focus on the hosting and curation of characterised SNPs that have been validated on a range of genotyping platforms, ensuring these SNPs can be quickly disseminated to plant breeding companies and scientists. The site has been designed to display SNP data in a way that is intuitive to its end users and accessible in a wide range of formats (such as excel spreadsheets), in response to the requirements of breeders who may not have access to the bioinformatics skills needed to parse information from more specialized formats such as variant call format (VCF). Users can download files in VCF format from the site via the download sections for each of the genotyping platforms (see http://www.cerealsdb.uk.net/cerealgenomics/CerealsDB/KASP_SNPs_070616.vcf).

Since its inception, CerealsDB has maintained a policy of making its data freely available to the public without restrictions or intellectual property rights. Users of CerealsDB are not required to register and there are no areas of the site that are password protected. The inclusion of web services means that data can automatically flow from CerealsDB to other databases, integrating resources and expanding the user base. Potential developments for CerealsDB could include the addition of annotations from other sources such as haplotype and phenotypic information currently being generated, however this would clearly need to integrate with the sites simple functionality to avoid becoming overly complicated and more importantly it should aim to be distinct from existing wheat resources which are in danger of homogenisation.

An example case study is included here to illustrate how simple it is to use the website. A user wanting to search for SNPs linked to disease resistance genes (these play a major role in plant responses to pathogens and pests and are of major interest to plant breeders) would start by selecting ‘Functional SNPs’ from the search page (http://www.cerealsdb.uk.net/cerealgenomics/CerealsDB/funcSNPs_select.php) and would then enter the term ‘disease’ in the ‘search term’ field. This would produce a list of SNPs linked to disease resistance based on the annotation given to the contig in which the SNP is located. Some of these SNPs, such as BS00023108, will already have an assay designed for them and the primer sequences can be downloaded. Entering the SNP’s BS code in the ‘Quick search’ box in the right hand menu, reveals that the SNP is available in the KASP and Axiom platforms and includes information such as KASP marker primer sequences that have been validated for this SNP, along with the contig sequence for BC000011796 (with SNP highlighted in red) and a chromosomal location on 7D. Should the user require additional detail, a simple click on the UniProt link takes them through to that website for information on gene ontologies and domains.

## Conclusions

CerealsDB version 3.0 site represents a considerable advance on previous versions, and includes large datasets of SNPs that have been characterised on a wide range of genotyping platforms. A subset of these SNPs have added value through BLAST annotation and the characterisation of functional SNPs. The site has additional functionality to help users to visualise the SNP genotyping data and the format of the site has been redesigned to help them find information more quickly. The data integration architecture provided by the Grassroots API will allow CerealsDB to seamlessly merge with wider networks of biological databases, and efforts to enforce current standards and formats (such as those suggested by the WheatIS consortium) have been adhered to, helping to guarantee their wider adoption by the community.

The CerealsDB website has a large user base across the globe as evidenced by the number of hits the site receives each month. The simplicity and functionality of the site along with the policy of releasing all data into the public domain without restriction has ensured that both plant breeding and wheat research communities continue to employ CerealsDB as an important tool in their efforts to develop new varieties of wheat that could help to solve global food shortages and deliver food security to the world’s burgeoning population.

## Availability and requirements

### Availability

CerealsDB can be accessed online from the following URL: http://www.cerealsdb.uk.net/cerealgenomics/CerealsDB/indexNEW.php. The SNP database is publicly and freely accessible, requiring no registration and with no restrictions on use. The non-synonymous and functional SNPs are available at the following URL: http://www.cerealsdb.uk.net/cerealgenomics/CerealsDB/funcSNPs_select.php.

### Technical requirements

It is recommended that one of the following browsers is used: Mozilla Firefox 3 on Linux, Mac OSX or Windows, Internet Explorer 8 on Windows, Safari 4 on Mac OsX or Windows, Chrome on Linux or Windows.

## Abbreviations

API, Application programming interface; BC, Bristol contig; BLAST, Basic local alignment search tool; BS, Bristol SNP; CPU, Central processing unit, CSS, Cascading style sheets; DArT, diversity array technology; ER, Entity-relationship; EST, Expressed sequence tag; FAO, Food and Agriculture Organisation of the United Nations; FAQ, Facts and questions; Gb, gigabytes; HTTP, Hypertext transfer protocol; ID, identification; JSON, Javascript object notation; MAS, marker-assisted selection; Mb, megabytes; NCBI, National Center for Biotechnology Information; NGS, Next generation sequencing; OS, Operating system; PHP, Hypertext pre-processor; RDMS, Relational database management system; REST, Representational state transfer; SNP, Single nucleotide polymorphism; TGAC, The genome analysis centre; URGI, Unité de Recherche Génomique Info; URI, Uniform Resource Identifier; URL, uniform resource locator; VCF, variant call format

## Additional file

Additional file 1: This file contains an initial web services request to CerealsDB in JSON, with the JSON response from CerealsDB containing a list of the services that the CerealsDB site provides (currently a Contig service and Search service). An example user query is also provided. (DOCX 560 kb)

## References

[1] Wilson ID, Barker GL, Beswick RW, Shepherd SK, Lu C, Coghill JA, Edwards D, Owen P, Lyons R, Parker JS, Lenton JR, Holdsworth MJ, Shewry PR, Edwards KJ (2004). A transcriptomics resource for wheat functional genomics. Plant Biotechnol J.

[2] Wilkinson PA, Winfield MO, Barker GLA, Allen AM, Burridge A, Coghill JA, Edwards KJ (2012). CerealsDB 2.0: an integrated resource for plant breeders and scientists. BMC Bioinformatics.

[3] Winfield MO, Allen AM, Burridge AJ, Barker GL, Benbow HR, Wilkinson PA, Coghill J, Waterfall C, Davassi A, Scopes G, Pirani A, Webster T, Brew F, Bloor C, King J, West C, Griffiths S, King I, Bentley AR, Edwards KJ (2015). High-density SNP genotyping array for hexaploid wheat and its secondary and tertiary gene pool. Plant Biotechnol J.

[4] The Wheat Initiative [http://www.wheatinitiative.org/]. Accessed on date 13 Jun 2016.

[5] International Wheat Information System [http://wheatis.org/]. Accessed on date 13 Jun 2016.

[6] Cuppen E. Genotyping by allele-specific amplification (KASPar). Cold Spring Harb Protocols. 2007. pp 172–173. 10.1101/pdb.prot4841 21357174

[7] Wang S, Wong D, Forrest K, Allen A, Chao S, Huang BE, Maccaferri M, Salvi S, Milner SG, Cattivelli L, Mastrangelo AM, Whan A, Stephen S, Barker G, Wieseke R, Plieske J, Lillemo M, Mather D, Appels R, Dolferus R, Brown-Guedira G, Korol A, Akhunova AR, Feuillet C, Salse J, Morgante M, Pozniak C, Luo MC, Dvorak J, Morell M, Dubcovsky J, Ganal M, Tuberosa R, Lawley C, Mikoulitch I, Cavanagh C, Edwards KJ, Hayden M, Akhunov E, IWGSC (2014). Characterization of polyploid wheat genomic diversity using a high-density 90,000 SNP array. Plant Biotechnol J.

[8] Allen AM, Barker GL, Berry ST, Coghill JA, Gwilliam R, Kirby S, Robinson P, Brenchley RC, D’Amore R, McKenzie N, Waite D, Hall A, Bevan M, Hall N, Edwards KJ (2011). Transcript-specific, single-nucleotide polymorphism discovery and linkage analysis in hexaploid bread wheat (*Triticum aestivum L*.). Plant Biotechnol J.

[9] Allen AM, Barker GLA, Wilkinson PA, Burridge AJ, Winfield MO, Coghill JA, Uauy C, Griffiths S, Jack P, Berry S, Werner P, Melichar J, McDougall J, Gwilliam R, Robinson P, Edwards KJ (2012). Discovery and development of exome-based, co-dominant single nucleotide polymorphism markers in hexaploid wheat (*Triticum aestivum L*.). Plant Biotechnology J.

[10] Kersey PJ, Allen JE, Armean I, Boddu S, Bolt BJ, Carvalho-Silva D, Christensen M, Davis P, Falin LJ, Grabmueller C, Humphrey J, Kerhornou A, Khobova J, Aranganathan NK, Langridge N, Lowy E, McDowall MD, Maheswari U, Nuhn M, Ong CK, Overduin B, Paulini M, Pedro H, Perry E, Spudich G, Tapanari E, Walts B, Williams G, Tello-Ruiz M, Stein J, Wei S, Ware D, Bolser DM, Howe KL, Kulesha E, Lawson D, Maslen G, Staines DM (2016). Ensembl Genomes 2016: more genomes, more complexity. Nucleic Acids Res.

[11] Altschul SF, Gish W, Miller W, Myers EW, Lipman DJ (1990). Basic local alignment search tool. J Mol Biol.

[12] Brenchley R, Spannagl M, Pfeiffer M, Barker GLA, D’Amore R, Allen AM, McKenzie N, Kramer M, Kerhornou A, Bolser D, Kay S, Waite D, Trick M, Bancroft I, Gu Y, Huo N, Luo MC, Sehgal S, Gill B, Kianian S, Anderson O, Kersey P, Dvorak J, McCombie WR, Hall A, Mayer MFX, Hall N, Edwards KJ, Bevan MW, Hall N (2012). Analysis of the bread wheat genome using whole-genome shotgun sequencing. Nature.

[13] Chapman JA, Mascher M, Buluç A, Barry K, Georganas E, Session A, Strnadova V, Jenkins J, Sehgal S, Oliker L, Schmutz J, Yelick KA, Scholz U, Waugh R, Poland JA, Muehlbauer GJ, Stein N, Rokhsar DS (2015). A whole-genome shotgun approach for assembling and anchoring the hexaploid bread wheat genome. Genome Biol.

[14] Milne I, Shaw P, Stephen G, Bayer M, Cardle L, Thomas WTB, Flavell AJ, Marshall D (2010). Flapjack – graphical genotype visualization. Bioinformatics.

[15] Grassroots Infrastructure project [https://wheatis.tgac.ac.uk] Accessed on date 13 Jun 2016.

[16] Shihab HA, Gough J, Cooper DN, Stenson PD, Barker GLA, Edwards KJ, Day INM, Gaunt TR (2013). Predicting the functional, molecular, and phenotypic consequences of amino acid substitutions using Hidden Markov Models. Hum Mutat.

[17] The UniProt Consortium (2015). UniProt: a hub for protein information. Nucleic Acids Res.

[18] Kersey PJ, Allen JE, Christensen M, Davis P, Falin LJ, Grabmueller C, Hughes DS, Humphrey J, Kerhornou A, Khobova J (2014). Ensembl Genomes 2013: scaling up access to genome-wide data. Nucleic Acids Res..

[19] The Wheat Portal [http://wheat-urgi.versailles.inra.fr/]. Accessed on date 13 Jun 2016.

[20] Matthews D, Carollo VL, Lazo GR, Anderson OD (2003). GrainGenes, the genome database for small-grain crops. Nucl Acids Res.

[21] Mayer KFX, Rogers J, Doležel J, Pozniak C, Eversole K, Feuillet C, Gill B, Friebe B, Lukaszewski AJ, The International Wheat Genome Sequencing Consortium (2014). A chromosome-based draft sequence of the hexaploid bread wheat (*Triticum aestivum*) genome. Science.

[22] Jordan KW, Wang S, Lun Y, Gardiner L-J, MacLachlan R, Hucl P, Wiebe K, Wong D, Forrest KL, Sharpe AG (2015). A haplotype map of allohexaploid wheat reveals distinct patterns of selection on homoeologous genomes. Genome Biol.

