# Supplementary material for: CerealsDB 3.0: expansion of resources and data integration
Source: BMC Bioinformatics. 2016 Jun 24;17:256. doi: 10.1186/s12859-016-1139-x (PMC4919907; doi:10.1186/s12859-016-1139-x)
Supplement: Additional file 1: — This file contains an initial web services request to CerealsDB in JSON, with the JSON response from CerealsDB containing a list of the services that the CerealsDB site provides (currently a Contig service and Search service). An example user query is also provided. (DOCX 560 kb) [file 12859_2016_1139_MOESM1_ESM.docx]

**Supplementary data**

**Initial JSON request to CerealsDB to retrieve a description of available web services:**

{

"operations": {

"operation_id": 0

}

}

**JSON response from CerealsDB listing available web services:**

{

"schema_version": 0.1,

"provider": {

"name": "CerealsDB",

"description": "A service to give information about SNP markers; e.g., the sequence upon which they are based, obtain primers used for their identification, identify the haplotypes of common UK varieties.",

"url": "http://www.cerealsdb.uk.net/cerealgenomics/cgi-bin/parse_json_v3.cgi"

},

"services": {

"path": "Contig service",

"summary": "A service to obtain contig information",

"description": "A service to obtain contig information using SNP names, contig names or generic search terms",

"operations": [

{

"operationId": "Contigservice",

"summary": "An operation to obtain contig information using SNP or Contig names",

"description": "An operation to obtain contig information using SNP or Contig names",

"parameter_set": {

"parameters": [

{

"param": "SNP_id",

"name": "SNP id",

"default": "BS00003643",

"current_value": "BS00003643",

"type": "string",

"tag": 1,

"wheatis_type": 11,

"description": "The SNP name (a 10 character alphanumeric beginning with 'BS' or 'BA'; e.g., BS00010624 or BA0033400) to find the contig to which it maps and all other putative SNPs, validated or not, that have been mapped to it. SNP names (IDs) may take different forms depending on the platform on which the assay was developed"

},

{

"param": "contig_name",

"name": "Contig name",

"default": "",

"current_value": "",

"type": "string",

"tag": 2,

"wheatis_type": 11,

"description": "The contig name (e.g. BC000000280)"

}

]

}

}, {

"operationId": "Search service",

"summary": "An operation to obtain contig information using generic search terms",

"description": "An operation to obtain contig information generic search terms",

"parameter_set": {

"parameters": [

{

"param": "Search_term",

"name": "Search term",

"default": "",

"current_value": "",

"type": "string",

"tag": 3,

"wheatis_type": 11,

"description": "Any keywords (e.g., 'disease', 'cold') "

}

]

}

}

]

}

}

**User query to CerealsDB in JSON format to extract data:**

{

"services": [{

"service": "Contigservice",

"run": true,

"parameter_set": {

"parameters": [{

"param": "contig_name",

"current_value": "BC000000118",

"tag": 2,

"type": "string",

"wheatis_type": 11,

"level": 7,

"concise": true

}, {

"param": "SNP_id",

"current_value": "BS00003644",

"tag": 1,

"type": "string",

"wheatis_type": 11,

"level": 7,

"concise": true

}]

}

}, {

"run": true,

"services": "Searchservice",

"parameter_set": {

"parameters": [{

"param": "Search_term",

"current_value": "rht",

"tag": 3

}]

}

}]

}

**Supplementary Figure S1.**

**
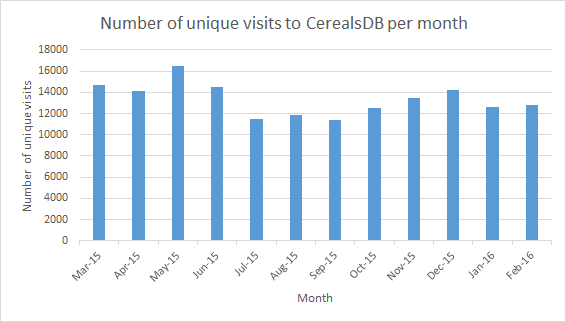
**

**Supplementary Figure S2.**

**
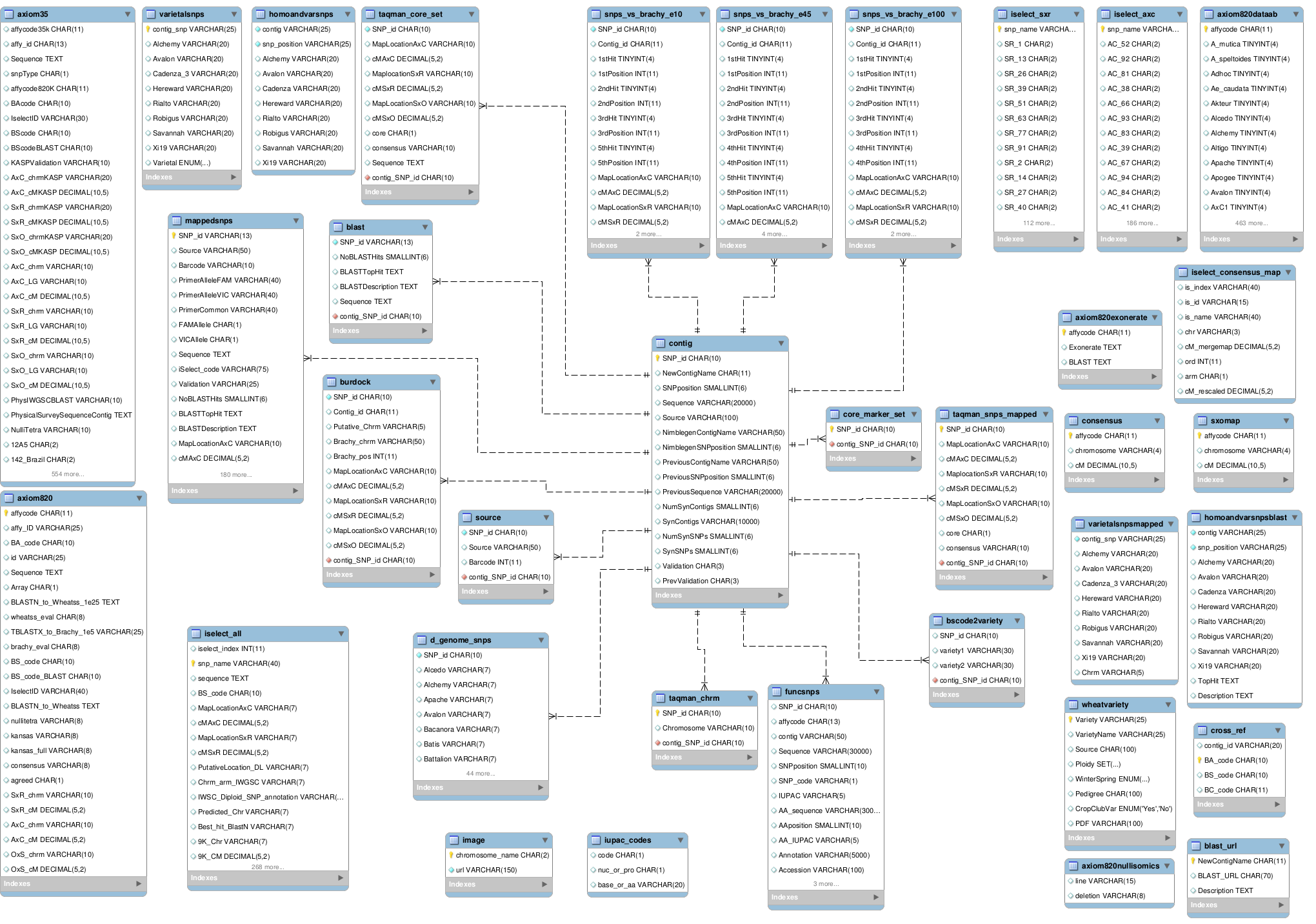
**
